# Acetoin: MAK-Begründung

**DOI:** 10.34865/mb51386d11_1or

**Published:** 2026-03-30

**Authors:** Andrea Hartwig, MAK Commission

**Affiliations:** 1 Institut für Angewandte Biowissenschaften, Abteilung Lebensmittelchemie und Toxikologie, Karlsruher Institut für Technologie (KIT) Adenauerring 20a, Geb. 50.41 76131 Karlsruhe Deutschland; 2 Ständige Senatskommission zur Prüfung gesundheitsschädlicher Arbeitsstoffe, Deutsche Forschungsgemeinschaft, Kennedyallee 40, 53175 Bonn, Deutschland. Weitere Informationen: Ständige Senatskommission zur Prüfung gesundheitsschädlicher Arbeitsstoffe | DFG

**Keywords:** Acetoin, MAK-Wert, maximale Arbeitsplatzkonzentration, Reizwirkung, Entzündung, Respirationstrakt, Hautresorption, Spitzenbegrenzung

## Abstract

The German Senate Commission for the Investigation of Health Hazards of Chemical Compounds in the Work Area (MAK Commission) summarized and evaluated the data for acetoin [513-86-0] to derive an occupational exposure limit value (maximum concentration at the workplace, MAK value) considering all toxicological end points. Relevant studies were identified from a literature search. A NOAEC of 400 ml/m^3^ for inflammatory effects induced by acetoin in the respiratory tract was obtained from a study with 3-month inhalation exposure of rats. To prevent these inflammatory effects, a MAK value of 50 ml/m^3^ has been derived for acetoin and the substance has been assigned to Peak Limitation Category II with an excursion factor of 2. There are no prenatal developmental toxicity studies of acetoin. Therefore, acetoin has been assigned to Pregnancy Risk Group D. The substance is not genotoxic in vitro and in vivo. A long-term carcinogenicity study has not been performed. According to skin absorption models, percutaneous absorption is expected to contribute significantly to systemic toxicity. Therefore, acetoin has been designated with “H”. A sensitizing potential is not expected based on the data available.

**Table:** 

**MAK-Wert (2025)**	**50 ml/m^3^ (ppm) ≙ 180 mg/m^3^**
**Spitzenbegrenzung (2025)**	**Kategorie II, Überschreitungsfaktor 2**
	
**Hautresorption (2025)**	**H**
**Sensibilisierende Wirkung**	**–**
**Krebserzeugende Wirkung**	**–**
**Fruchtschädigende Wirkung (2025)**	**Gruppe D**
**Keimzellmutagene Wirkung**	**–**
	
**BAT-Wert**	**–**
	
Synonyma	Acetylmethylcarbinol Butan-2-ol-3-on 3-Hydroxy-2-butanon 1-Hydroxyethylmethylketon
Chemische Bezeichnung (IUPAC-Name)	3-Hydroxybutan-2-on
CAS-Nr.	513-86-0 53584-56-8 ((*R*)-Acetoin) 78183-56-9 ((*S*)-Acetoin)
Formel	CH_3_–CH(OH)–CO–CH_3_
	C_4_H_8_O_2_
Molmasse	88,11 g/mol
Schmelzpunkt	15 °C (Monomer, IFA 2024) 90 °C (Dimer, IFA 2024)
Siedepunkt	128,4 °C bei 1021,5 hPa (ECHA 2019) 148 °C bei 1013 hPa (NCBI 2024)
Dichte bei 20 °C	1,01 g/cm^3^ (IFA 2024)
Dampfdruck bei 20 °C	7,6 hPa (Efron und Blom 1947) 3,6 hPa (ber.; NCBI 2024)
log K_OW_	0,1 (ECHA 2019) –0,36 (ber.; NCBI 2024)
Löslichkeit	1000 g/l Wasser (ECCC und Health Canada 2019)
**1 ml/m^3^ (ppm) ≙ 3,656 mg/m^3^**	**1 mg/m^3^ ≙ 0,274 ml/m^3^ (ppm)**
	
Hydrolysestabilität	k. A.
Stabilität	Bei Raumtemperatur langsame Dimerisierung zu 2,3,5,6-Tetramethyl-1,4-dioxan-2,5-diol (CAS-Nr. 23147-57-1) (siehe Abbildung 1), in Anwesenheit von Methanol und unter Lichteinfluss kann Acetoin zu Diacetyl oxidieren (Pendergrass 2004)
Verwendung	Geschmacksstoff, Duftstoff (NCBI 2024), Butteraroma, Zusatz für E-Zigaretten, Duftstoffträger bei der Herstellung von Parfüms und Essenzen (NTP 2023)

Acetoin ist in der Natur weit verbreitet und wird von Mikroorganismen, Pflanzen und Tieren produziert. Natürlich vorkommendes Acetoin trägt zu den Aromen von Butter, Honig, Kakao, Früchten, Gemüse und Mehlen bei. Fast alle alkoholischen Getränke enthalten Acetoin, da es bei der Hefe- oder Bakteriengärung entsteht. Acetoin ist eine flüchtige Verbindung, die auch in frischem menschlichen Schweiß (9,5 %) vorhanden ist (NTP 2023).

Handelsübliches Acetoin besteht zumeist aus einer Mischung von Monomer und Dimer (Vas et al. 2019). Die Dimerisierung ist in Abbildung 1 dargestellt.

**Abb. 1:**
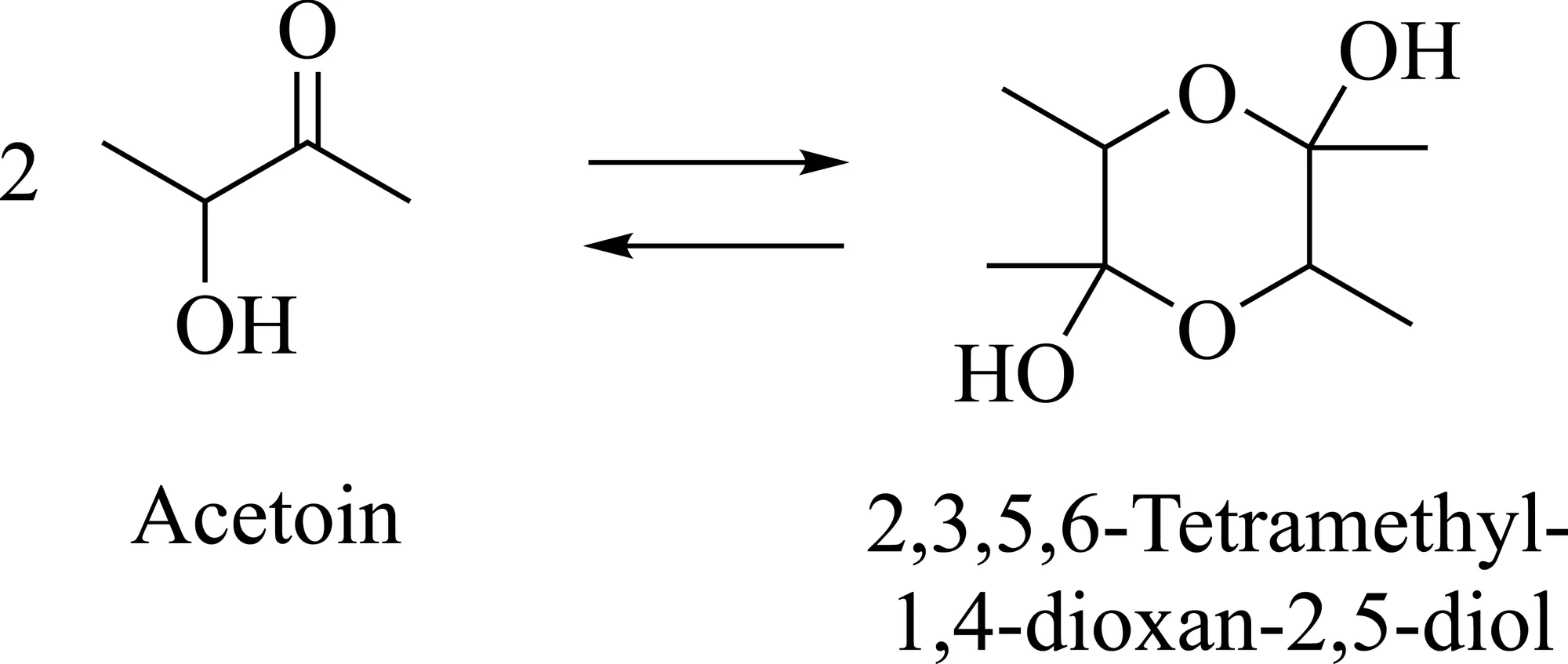
Dimerisierung von Acetoin

Die Zugabe von Acetoin als synthetischer Geschmacksstoff zur Nahrung ist in den USA als sicher eingestuft (generally recognized as safe, GRAS), dies wurde von der FDA (Food and Drug Administration) festgelegt (21 CFR 182.60) (NTP 2023).

Acetoin in E-Liquids kann in Diacetyl umgewandelt werden und ist daher eine unvermeidliche Quelle einer Diacetyl-Belastung für E-Zigaretten-Raucher (Vas et al. 2019).

## 1 Allgemeiner Wirkungscharakter

Acetoin wirkt an der Kaninchenhaut nach 24-stündiger okklusiver Applikation mäßig reizend und im Reconstructed human Epidermis (RhE)-Test in vitro nicht reizend. Der Stoff verursacht in vitro schwere Schäden an der Hornhaut des Rinderauges.

Nach 13-wöchiger Ganzkörperexposition gegen 800 ml Acetoin/m^3^ treten Entzündungen im Larynx, Metaplasien und Infiltrationen in der Lunge sowie Nephropathie bei den männlichen und in geringerem Umfang bei den weiblichen Ratten auf. Alle Effekte sind minimal bis gering ausgeprägt.

Acetoin ist nicht mutagen in bakteriellen Mutagenitätstests und zeigt keine genotoxischen Effekte in vivo im Mikronukleustest bei Ratte und Maus.

Es liegen keine Hinweise auf eine sensibilisierende Wirkung von Acetoin vor.

Es gibt keine Daten zur Kanzerogenität und Entwicklungstoxizität.

## 2 Wirkungsmechanismus

Acetoin ist weniger reaktiv als sein oxidativer Metabolit Diacetyl. So treten nach inhalativer Exposition von Mäusen gegen 800 ml Acetoin/m^3^ keine adversen Effekte im Atemtrakt auf, während es mit 50 ml Diacetyl/m^3^ zu Entzündungen in Nase und Lunge kommt (Abschnitt 5.2.1). Dieser Unterschied ist darin begründet, dass die zwei benachbarten Carbonylgruppen die Delokalisation des Elektrons des entstehenden Radikalanions begünstigen, wodurch Diacetyl an Biomoleküle binden und mit primären Aminen konjugierte Imine bilden kann (Hartwig 2015), was mit Acetoin nicht möglich ist.

Die Interleukin (IL)-8-Freisetzung nach Inkubation einer humanen bronchialen Epithelzelllinie mit Acetoin gibt einen Hinweis auf eine mögliche proinflammatorische Wirkung (Abschnitt 5.8; Gerloff et al. 2017), die in vivo erst bei hohen Konzentrationen beobachtet wurde (Abschnitt 5.2.1; NTP 2023).

Acetoin war nur in einem von 320 untersuchten In-vitro-Tests der ToxCast-Assay-Suite positiv: Die beobachtete Induktion der Enzymaktivität von CYP2D6 (human) im zellfreien System wurde nur bei der höchsten Konzentration von 10 µM erreicht (US EPA 2025).

## 3 Toxikokinetik und Metabolismus

### 3.1 Aufnahme, Verteilung, Ausscheidung

Es liegen keine Daten zur Toxikokinetik von Acetoin nach **inhalativer** Aufnahme vor.

Der Blut:Luft-Verteilungskoeffizient errechnet nach Buist et al. (2012) liegt bei 15 979.

An anatomisch akkuraten Modellen der Nasengänge von Mensch und Ratte wurde mithilfe eines „computational fluid dynamics“-Modells die Aufnahme im respiratorischen Trakt simuliert. Nach Exposition gegen 10 ml Acetoin/m^3^ wurde eine Aufnahme im nasalen und oberen Atembereich des humanen Modells von 32,5 % und des Ratten-Modells von 73,6 % berechnet. Die Aufnahme von Diacetyl nach Exposition gegen 10 ml/m^3^ lag im oberen Atembereich mit 8,7 % (human) und 30,9 % (Ratte) deutlich niedriger. Die Aufnahme von Acetoin in den gesamten Atemtrakt betrug 85,9 % (human) und 93 % (Ratte). Die Autoren führen die im Vergleich zum Diacetyl höhere Aufnahme von Acetoin in den oberen Atemtrakt auf seine bessere Wasserlöslichkeit zurück (Schroeter et al. 2025).

Männlichen Wistar-Ratten wurde eine **orale** Einzeldosis von 5 mmol Acetoin/kg Körpergewicht (ca. 440 mg Acetoin/kg KG) verabreicht. Eine Stunde nach der Verabreichung konnte Acetoin in Leber (ca. 60 nmol/g), Nieren (ca. 20 nmol/g) und Gehirn (ca. 140 nmol/g) bestimmt werden. Das Reduktionsprodukt 2,3-Butandiol wurde in Konzentrationen von ca. 1,8–3,1 µmol/g in den drei Geweben nachgewiesen (Kontrollwerte < 0,07 µmol/g). Eine Oxidation zu Diacetyl erfolgte in weit geringerem Maße mit Konzentrationen von ca. 17 nmol/g in der Leber, ca. 6 nmol/g in den Nieren und ca. 16 nmol/g im Gehirn (k. w. A.; Otsuka et al. 1996). Alle angegebenen Konzentrationen in den Geweben wurden aus einem Diagramm abgelesen.

Nach **oraler** Gabe an Ratten wurde Acetoin zu 2,3-Butandiol reduziert und glucuronidiert mit dem Urin ausgeschieden oder weiter metabolisiert und als CO_2_ abgeatmet (siehe Abschnitt 3.2; NCBI 2024).

Von Ratten und Mäusen werden **oral** verabreichte aliphatische alpha-Diketone rasch aus dem Magen-Darm-Trakt resorbiert. Ähnliches dürfte für Acetoin zutreffen (ECCC und Health Canada 2019).

Nach der wiederholten **intraperitonealen** Injektion von kleinen Mengen einer 30%igen wässrigen Acetoinlösung (Dosierung nicht angegeben) an Ratten führten Konzentrationen im Blut von 2270 und 2510 mg Acetoin/l (durchschnittlich 2350 mg/l) zum Verlust des Aufrichtungsreflexes und von 7420 und 7700 mg Acetoin/l (durchschnittlich 7540 mg/l) zu Atemstillstand. Nach intraperitonealer Injektion von Ethanol wurden Alkoholkonzentrationen im Blut der Ratten zwischen 2880 und 3120 mg/l (Verlust des Aufrichtungsreflexes) bzw. 9000 und 9520 mg/l (Atemstillstand) benötigt. Damit wirkte Acetoin etwas stärker als Ethanol. Bei kombinierter Verabreichung von Acetoin und Ethanol addierte sich die Wirkung der beiden Chemikalien (Greenberg 1943).

Nach einer **intrakardialen** Injektion von [2,3-^14^C]-Acetoin an Sprague-Dawley-Ratten wurde ^14^CO_2_ in der Ausatemluft nachgewiesen. Nach 20 Stunden wurden bis zu 47,7 % ^14^CO_2_ gemessen. Die mittlere 12-Stunden-CO_2_-Produktion nach **intraperitonealer** Injektion lag bei 15 % (Gabriel et al. 1972).

Experimente zur **dermalen** Resorption von Acetoin liegen nicht vor. Für eine gesättigte wässrige Lösung berechnen sich mit dem Modell von Fiserova-Bergerova et al. (1990) und dem Algorithmus des IH SkinPerm-Modells (Tibaldi et al. 2014) bei Annahme eines log K_OW_ von 0,1 Fluxe von 3,755 bzw. 0,858 mg/cm^2^ und Stunde. Unter der Annahme einer einstündigen Exposition von 2000 cm^2^ Hautoberfläche würde dies Aufnahmemengen von 7509 bzw. 1720 mg entsprechen. Eine entsprechende Berechnung unter Annahme eines log K_OW_ von –0,36 führt zu Fluxen von 1705 bzw. 447 mg/cm^2^ und Aufnahmemengen von 3412 bzw. 895 mg.

**Fazit**: Es liegen keine Studien zur Toxikokinetik von Acetoin nach inhalativer oder dermaler Aufnahme vor. Nach oraler Aufnahme im Tierversuch wird Acetoin rasch aus dem Magen-Darm-Trakt resorbiert und nachfolgend, wie sein Reduktionsprodukt 2,3-Butandiol, systemisch mit dem Blut verteilt. Die Ausscheidung erfolgt via glucuronidierter Metaboliten mit dem Urin und durch Abatmung von Kohlenstoffdioxid nach weiterer Metabolisierung. Beim Menschen würde eine einstündige dermale Exposition von 2000 cm^2^ Hautoberfläche zu Aufnahmemengen von 895–7509 mg führen.

### 3.2 Metabolismus

Acetoin und Diacetyl sind beim Menschen endogen produzierte Stoffe. Sie werden gebildet, wenn Pyruvat durch Pyruvat-Decarboxylase reduktiv in Acetoin und oxidativ in Diacetyl umgewandelt wird (Gabriel et al. 1972). Es wurden mittlere Nüchternblutkonzentrationen beim Menschen von etwa 1 mg Acetoin/l Blut berichtet (WHO 1999).

In einer Probandenstudie zur Ethanolwirkung wurde bei zehn Kontrollpersonen auch Acetoin im Plasma bestimmt und eine mittlere Konzentration von 2,2 µM (ca. 193,84 µg/l) gemessen. Die Konzentrationen von Diacetyl und Butan-2,3-diol lagen bei 0,25–0,75 µM (ca. 21,52–64,57 µg/l) bzw. 5–10 µM (ca. 450,6–901,2 µg/l) im Plasma (Otsuka et al. 1999). Die hier aufgeführten Konzentrationen im Blut weisen darauf hin, dass der reduktive Stoffwechselweg überwiegt.

Acetoin wurde Ratten entweder oral in einer 3–4%igen Lösung oder subkutan in einer 20%igen Lösung verabreicht, wobei mehrere Dosen gleichmäßig über den Tag verteilt gegeben wurden. Acetoin wurde kaum mit dem Urin ausgeschieden und es wurde kein Diacetyl nachgewiesen; das wichtigste Ausscheidungsprodukt war 2,3-Butandiol, der Rest wurde vollständig metabolisiert. Einem Hund wurde über zwei Monate eine Gesamtdosis von 78 g Acetoin verabreicht, sowohl oral in einer 3–4%igen Lösung als auch subkutan in einer 20%igen Lösung. Der Urin wurde von Beginn der Behandlung bis 40 Stunden nach der letzten Dosis unter Toluolatmosphäre gesammelt. 2,3-Butandiol war das Hauptausscheidungsprodukt mit dem Urin, das 5–25 % der verabreichten Dosis ausmachte. Der Rest der Dosis wurde fast vollständig metabolisiert. Es konnten minimale Mengen Acetoin im Urin nachgewiesen werden (k. w. A.; NCBI 2024). In einer anderen Studie wurde 2,3-Butandiol leicht mit Glucuronsäure konjugiert (Westerfeld und Berg 1943).

Der Metabolismus von Acetoin, Diacetyl und 2,3-Butandiol, die aus Acetaldehyd und Pyruvat entstehen, wurde anhand von Rattenleberhomogenat, Leberperfusion und In-vivo-Versuchen mit Wistar-Ratten quantitativ untersucht. Die orale Gabe von 5 mmol Acetoin/kg KG (ca. 440 mg Acetoin/kg KG) an männliche Wistar-Ratten führte eine Stunde nach der Verabreichung zu hohen Konzentrationen in Leber (ca. 60 nmol/g), Nieren (ca. 20 nmol/g) und Gehirn (ca. 135 nmol/g), damit trat die höchste Konzentration im Gehirn auf. Das Reduktionsprodukt 2,3-Butandiol wurde ebenfalls in Leber, Nieren und Gehirn nachgewiesen. Die Reduktion von Acetoin zu 2,3-Butandiol erfolgte überwiegend in der Leber und erforderte NADH. Die organspezifische Reduktaseaktivität war am größten in der Leber und am geringsten im Gehirn. Im Urin wurde 2,3-Butandiol konjugiert als Glucuronid identifiziert. Nach 10-minütiger Inkubation von **Leberhomogenat** (Ratte) mit Acetoin (10 nmol) konnte kein Diacetyl nachgewiesen werden. Etwa 70 % waren zu 2,3-Butandiol reduziert worden. Eine Stunde nach Acetoingabe per **Leberperfusion** an Ratten wurden in der Leber (nach Homogenisierung von 1 g) und in der Perfusatlösung Acetoin und in dreifach höherer Menge 2,3-Butandiol nachgewiesen (Otsuka et al. 1996).

Nach Inkubation von Ratten- und Kaninchen-**Leberhomogenaten** mit [2,3-^14^C]-Acetoin konnten anhand der radioaktiven Markierung > 95 % als ein Gemisch von Stereoisomeren des 2,3-Butandiols, aber kein Acetoin, wiedergefunden werden und es entstand kaum CO_2_. Die Reduktion des Acetoins wurde durch die Acetoin-Reduktase katalysiert und benötigte dafür die Coenzyme NAD^+^/NADH (Gabriel et al. 1971). Die Reduktion von Ketonen wird durch die Alkoholdehydrogenase und NADPH-abhängige zytosolische Carbonylreduktasen katalysiert (WHO 1999).

In Tabelle 1 sind die endogenen Diacetyl- und Acetoin-Gehalte in Geweben, Plasma und Urin von fünf männlichen Wistar-Ratten dargestellt. Es wurden nur geringe Unterschiede zwischen den Acetoin- und Diacetyl-Konzentrationen gemessen, nur im Gehirn und im Urin war die Acetoin-Konzentration deutlich höher (Otsuka und Ohmori 1992).

**Tab. 1:** Endogene Acetoin- und Diacetyl-Konzentrationen in verschiedenen Geweben sowie im Plasma und Urin der Ratte, Mittelwerte ± Standardabweichung (Otsuka und Ohmori 1992)

**Gewebe**	**Diacetyl nmol/g**	**Acetoin nmol/g**
Leber	3,40 ± 0,95	3,62 ± 0,42
Herz	2,18 ± 0,45	1,19 ± 0,32
Nieren	2,64 ± 0,29	1,38 ± 0,53
Muskel	0,67 ± 0,11	0,42 ± 0,19
Gehirn	2,36 ± 0,31	5,76 ± 0,81
Plasma	3,29 ± 0,30 nmol/ml	1,60 ± 0,22 nmol/ml
Urin	1,78 ± 0,38 nmol/mg Kreatinin	2,94 ± 0,21 nmol/mg Kreatinin

**Abb. 2:**
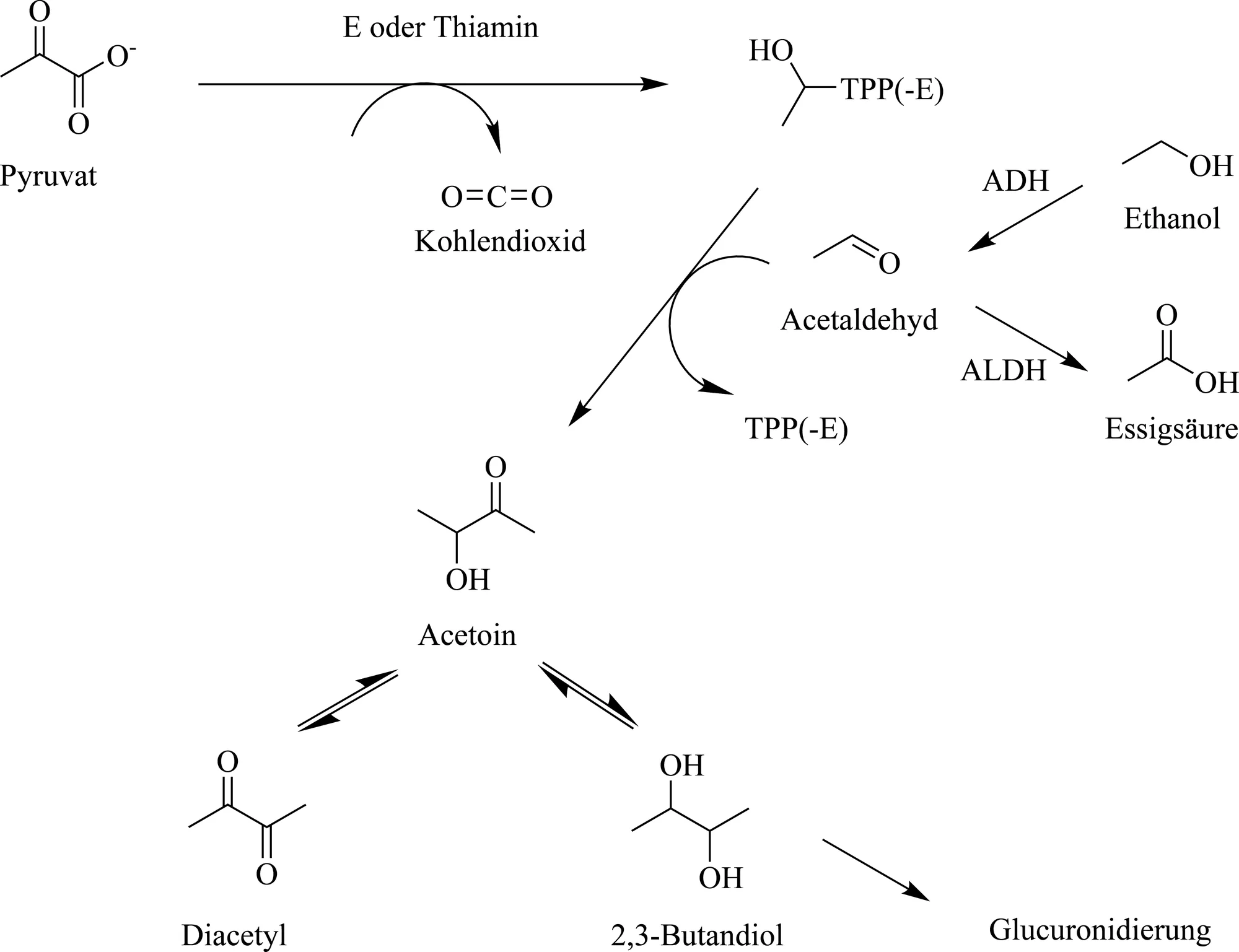
Metabolismusschema nach Otsuka (1996). Übersetzt und reproduziert mit freundlicher Genehmigung von Oxford University Press.

ADH: Alkoholdehydrogenase; ALDH: Aldehyddehydrogenase; E: Pyruvatdehydrogenase-Komplex; TPP: Thiaminpyrophosphat

Gemäß der vorliegenden Daten kann das in Abbildung 2 dargestellte Metabolismusschema für Acetoin angenommen werden.

#### 3.2.1 Fazit

Acetoin ist ein endogener Stoff. Acetoin wird von Ratten oral gut aufgenommen und in der Leber schnell und fast vollständig zu CO_2_ und 2,3-Butandiol reduziert. Die Ausscheidung erfolgt über die Ausatemluft als CO_2_ oder als 2,3-Butanol-Glucuronid im Urin. Eine Oxidation zu Diacetyl findet nur in sehr geringem Ausmaß statt. Damit kann ausgeschlossen werden, dass es nach Acetoin-Exposition zu einem wesentlichen Anstieg der Diacetyl-Konzentrationen im Gewebe kommt, der zu adversen Effekten führen könnte.

## 4 Erfahrungen beim Menschen

Die Geruchsschwelle von Acetoin ist nicht bekannt. Der Geruch ist angenehm und die Geruchsstärke hoch (TGSC Information System 2025). Durch die eher angenehme Geruchsqualität und Unsicherheiten bei der tatsächlichen Geruchsschwelle ist eine Markierung der Substanz mit der Fußnote „Auch bei Einhaltung des MAK-Werts sind im Einzelfall „Geruchs-assoziierte“ Symptome nicht auszuschließen“ nicht erforderlich, da die relevanten Kriterien (DFG 2025; van Thriel et al. 2023) nicht erfüllt sind.

### 4.1 Einmalige Exposition

Hierzu liegen keine Daten vor.

### 4.2 Wiederholte Exposition

Nach inhalativer Exposition gegen Butteraromastoffgemische mit Acetoin und Diacetyl traten bei Beschäftigten der Lebensmittelindustrie irreversible Atemwegsobstruktionen, u. a. Bronchiolitis obliterans, auf. Diese Erkrankungen wurden ausschließlich auf Diacetyl zurückgeführt. Die Konzentrationen für Acetoin lagen im Bereich von < 0,004 bis maximal 1,07 ml/m^3^ und für Diacetyl von 0,08 bis 37,8 ml/m^3^ (Hartwig 2015).

### 4.3 Wirkung auf Haut und Schleimhäute

Eine 10%ige Acetoin-Zubereitung in Vaseline verursachte in einem 48-stündigen okklusiven Patchtest keine Reizung (k. w. A.; Opdyke 1979).

### 4.4 Allergene Wirkung

#### 4.4.1 Hautsensibilisierende Wirkung

Ein Maximierungstest an 31 Freiwilligen mit 10 % Acetoin in Vaseline führte zu keinen Reaktionen (Epstein 1977 sekundär zitiert in Opdyke 1979).

#### 4.4.2 Atemwegssensibilisierende Wirkung

Es liegen keine gezielten Untersuchungen zur atemwegssensibilisierenden Wirkung von Acetoin vor.

In einem Popcorn-Herstellungsbetrieb entwickelten drei Mitarbeiter beruflich bedingtes Asthma, zwei davon möglicherweise auch Bronchiolitis obliterans. Eine Luftanalyse ergab eine Exposition gegen Diacetyl, Acetoin, 2-Nonanon, Acetaldehyd und weitere flüchtige organische Verbindungen. Als Ursache für die Erkrankung wird Diacetyl vermutet. Aufgrund der Mischexposition lassen sich aus den Untersuchungen keine Rückschlüsse auf eine atemwegssensibilisierende Wirkung von Acetoin ziehen (Sahakian et al. 2008).

### 4.5 Reproduktionstoxizität

Hierzu liegen keine Daten vor.

### 4.6 Genotoxizität

Hierzu liegen keine Daten vor.

### 4.7 Kanzerogenität

Hierzu liegen keine Daten vor.

## 5 Tierexperimentelle Befunde und In-vitro-Untersuchungen

### 5.1 Akute Toxizität

#### 5.1.1 Inhalative Aufnahme

Nach sechsstündiger Ganzkörperexposition gegen 0 oder 155 ml Acetoin/m^3^ wurden bei je sechs männlichen Sprague-Dawley(Hla:(SD)CVF)-Ratten, im Gegensatz zur Exposition gegen Diacetyl, keine Nekrosen im respiratorischen Epithel der Nase nachgewiesen (Cichocki et al. 2014; Hubbs et al. 2019).

Ganzkörperexpositionen gegen 0, 100 oder 150 ml Acetoin/m^3^ über einen Zeitraum von sechs Stunden führten bei je sieben männlichen Sprague-Dawley-Ratten, die 18 Stunden später für die Untersuchungen anästhesiert wurden, nicht zu einem Effekt im Methacholintest auf bronchiale Hyperreagibilität, in Tests auf Lungenwiderstand oder dynamischer Compliance (Zaccone et al. 2013).

#### 5.1.2 Orale Aufnahme

Die LD_50_ lag bei der Ratte über 5000 mg/kg KG (k. w. A.; Opdyke 1979).

#### 5.1.3 Dermale Aufnahme

Die LD_50_ lag bei Kaninchen über 5000 mg/kg KG (k. w. A.; Opdyke 1979).

#### 5.1.4 Subkutane, intravenöse und intraperitoneale Aufnahme

Eine einmalige subkutane oder intraperitoneale Gabe von 14 g Acetoin/kg KG an Ratten führte zum Verenden der Tiere mit Paralyse und Krämpfen (Konvulsion). Die halbe Dosis führte zu ähnlichen Symptomen, aber die Tiere erholten sich. Erste Symptome einer Paralyse traten nach subkutaner Gabe von ca. 3,5 g/kg KG auf, jedoch nicht mehr bei einer Dosis von ca. 1,75 g/kg KG (Westerfeld und Berg 1943).

Nach der intraperitonealen Injektion von kleinen Mengen einer 30%igen wässrigen Acetoinlösung (Dosierung nicht angegeben) an Ratten führten Konzentrationen im Blut von 2270 und 2510 mg Acetoin/l (durchschnittlich 2350 mg/l) zum Verlust des Aufrichtungsreflexes und von 7420 und 7700 mg Acetoin/l (durchschnittlich 7540 mg/l) zu Atemstillstand. Nach intraperitonealer Injektion von Ethanol wurden für die gleichen Wirkungen Ethanolkonzentrationen im Blut der Ratten zwischen 2880 und 3120 mg/l (Verlust des Aufrichtungsreflexes) bzw. 9000 und 9520 mg/l (Atemstillstand) benötigt. Damit wirkte Acetoin etwa 1,4-mal so stark wie Ethanol. Bei kombinierter Verabreichung von Acetoin und Ethanol addierte sich die Wirkung der beiden Chemikalien (Greenberg 1943). Die einmalige intraperitoneale Verabreichung von 5,5 mmol Acetoin/kg KG (ca. 500 mg/kg KG) an Ratten führte nicht zu narkotischen Effekten (Sprince et al. 1966).

Nach intraperitonealer Injektion von 3600 mg Acetoin/kg KG in 0,9%iger NaCl-Lösung an männliche Sprague-Dawley-Ratten wurde kein Einfluss auf den Sauerstoffverbrauch oder den Blutdruck beobachtet (Hohenegger et al. 1988).

Intravenöse Acetoin-Gaben an Katzen führten zu Veränderungen im Elektroenzephalogramm (EEG). Nach einer intraperitonealen Gabe traten bei Ratten als Haupteffekt Bewusstseinsstörungen auf (k. w. A.; Opdyke 1979).

### 5.2 Subakute, subchronische und chronische Toxizität

#### 5.2.1 Inhalative Aufnahme

In den nachfolgend beschriebenen Inhalations-Studien lag Acetoin als festes Dimer vor und wurde erhitzt, um das flüssige Monomer zu bilden, das zur inhalativen Exposition verwendet wurde. Ein Partikel-Detektor überprüfte durchgängig, dass keine Aerosole auftraten, die inhaliert werden könnten. Die Reinheit des Monomers betrug 99,4 % (NTP 2023).

In einer Vorstudie zur Drei-Monate-Studie wurden je fünf weibliche Wistar-Ratten (Crl:WI(Han)) und B6C3F1-Mäuse zwei Wochen und zwei (Ratten) bzw. drei Tage (Mäuse) an sechs Stunden pro Tag und fünf Tagen in der Woche gegen 0; 6,25; 25; 100; 400 oder 800 ml Acetoin/m^3^ ganzkörperexponiert. Es wurden keine Effekte beobachtet (NTP 2023).

In einer Untersuchung ähnlich der OECD-Prüfrichtlinie 413 wurden je zehn männliche und weibliche Wistar-Ratten (Crl:WI(Han)), an sechs Stunden pro Tag und fünf Tagen in der Woche, 13–14 Wochen lang gegen 0, 50, 100, 200, 400 oder 800 ml Acetoin/m^3^ ganzkörperexponiert. Das Blut wurde am 4., 23. und 93. Tag untersucht. Am 23. Tag, jedoch nicht am Studienende, traten statistisch signifikant verringerte Neutrophilenzahlen ab der niedrigsten Konzentration auf und Hämatokritwerte und Hämoglobin waren ab 100 ml/m^3^ verringert. Ein konzentrationsabhängiger Anstieg des Prozentsatzes unreifer Erythrozyten (% PCE) wurde bei männlichen Ratten ab 200 ml/m^3^ beobachtet, was auf eine leichte Störung der Erythropoese schließen lässt, die von den Autoren als nicht bedeutend bewertet wurde (vgl. Abschnitt 5.6.2). Bei den männlichen Ratten wurde ab 200 ml/m^3^ eine statistisch signifikante Abnahme der Lymphozytenzahlen (siehe Tabelle 3) am Studienende beobachtet, und verbunden damit sanken auch die Leukozytenzahlen (siehe Tabelle 4). Veränderte Harnstoff- und Kreatinin-Konzentrationen bei 800 ml/m^3^ werden von den Autoren als physiologische Antwort (Anpassung) auf reduzierte Futter- und Wasseraufnahme am Beginn der Expositionen zurückgeführt. Bei den männlichen Ratten der 800-ml/m^3^-Gruppe war das relative Lebergewicht statistisch signifikant erhöht (7 %). Eine histopathologische Untersuchung wurde nur bei allen Tieren der höchsten Konzentrationsgruppe durchgeführt. Bei einzelnen Tieren beider Geschlechter traten Infiltrationen, Metaplasien und Entzündungen im respiratorischen System (Lunge und Larynx) auf. Im Vergleich zu den Kontrolltieren lag eine gering erhöhte Nephropathie vor. Nach Ganzkörperexposition über einen Zeitraum von 13–14 Wochen (fünf Tage/Woche, sechs Stunden/Tag) von je zehn männlichen und weiblichen B6C3F1-Mäusen gegen 0, 50, 100, 200, 400 oder 800 ml/m^3^ wurden keine statistisch signifikanten Effekte beobachtet. Das Körpergewicht und die Nahrungsaufnahme waren bei beiden Spezies nicht verringert. Bei beiden Spezies wurden folgende Untersuchungen durchgeführt: Nekropsie, Organgewichtsmessungen, mikroskopische Gewebsuntersuchungen, komplette Histopathologie nur bei den Kontrolltieren und den Tieren der höchsten Expositionskonzentration, klinisch-chemische (nur Ratten) und hämatologische Untersuchungen. Die genauen Daten der Inhalationsstudien bei Ratte und Maus sind in Tabelle 2 dargestellt. In dieser Studie liegt die NOAEC laut der Autoren bei 800 ml/m^3^ (NTP 2023). Der statistisch signifikante Unterschied zwischen den Neutrophilenzahlen der männlichen Ratten im Vergleich zur Kontrolle ab 50 ml/m^3^ am 23. Expositionstag könnte auf die sehr hohen Neutrophilenzahlen der Kontrolltiere zurückzuführen sein. Dies verdeutlichen die in Tabelle 5 vergleichend dargestellten Neutrophilenzahlen der Kontrolltiere aus drei verschiedenen Studien des NTP. Betrachtet man die Anzahl der Neutrophilen am 23. Tag (absolute Zahlen), und setzt sie nicht ins Verhältnis zu den Kontrolltieren, ist keine Auffälligkeit bei den exponierten männlichen Ratten zu erkennen (Tabelle 6). Dasselbe gilt auch für die Werte von Hämatokrit und Hämoglobin am 23. Tag ab 100 ml/m^3^, was ebenfalls nicht als substanzbedingt angesehen wird. Das geringfügig erhöhte relative Lebergewicht wird von der Kommission nicht als advers angesehen, da es nur eine adaptive Anpassung an die Acetoin-Exposition ausdrückt. Die beobachtete Abnahme an Leukozyten (hier Lymphozyten und Monozyten) der männlichen Ratten bis zur höchsten Konzentration wird nicht von einer Gewichtsabnahme oder Veränderung des Thymus begleitet. Auch für das Knochenmark werden keine Effekte berichtet. Damit ist hier nicht von einer Immunsuppression auszugehen. Ob bereits bei Exposition gegen 400 ml/m^3^ histopathologisch als advers einzustufende Effekte auftraten, kann nicht bewertet werden, da es nicht untersucht wurde, weil schon bei 800 ml/m^3^ keine statistisch signifikant erhöhten Befunde festgestellt wurden.

Die Autoren der Studie des NTP (2023) weisen darauf hin, dass die statistisch signifikante Abnahme der Lymphozyten nach 93-tägiger Exposition gegen Acetoin ab 200 ml/m^3^ in einem Zusammenhang mit einem Stress-induzierten chronischen Anstieg der endogenen Glukokortikoide zu sehen sein könnte. Die Veränderungen der Leukozytenzahlen sind damit ihrer Meinung nach nicht substanzbedingt. Die Autoren verweisen auf das von Everds et al. (2013) beschriebene Stress-Leukogramm: Leukozyten, insbesondere Neutrophile, Lymphozyten und Eosinophile sind besonders sensitive Indikatoren von Stress. Längerfristiger Stress (d. h. Tage bis Wochen) kann zu einem Rückgang der Lymphozyten- und Eosinophilenzahlen führen, während die Neutrophilenzahlen zumeist erhöht, aber auch unverändert oder verringert sein können. Die Abnahme der Lymphozytenzahl infolge eines Glukokortikoidanstiegs ist bei Tierarten, bei denen Lymphozyten überwiegen, z. B. Ratten und Mäuse, ausgeprägter als die Zunahme der Neutrophilenzahl. Ebenso können die Retikulozytenzahlen, die Erythrozytenzahlen sowie Hämoglobin und Hämatokrit unverändert oder verringert sein. Der Zeitpunkt und das Ausmaß dieser Auswirkungen hängen vom Ausmaß und der Dauer des Stressors ab (Everds et al. 2013).

In der NTP-Studie (2023) waren die Eosinophilenzahlen bei den männlichen Ratten auf bis zu 69 % des Kontrollwerts reduziert, allerdings nicht statistisch signifikant. Insgesamt sind die hämatologischen Befunde mit einer Reaktion auf chronischen Stress kompatibel. Da die Kontrolltiere in der Studie des NTP (2023) den gleichen Haltungsbedingungen wie die exponierten Tiere ausgesetzt waren (Expositionskammer, fünf Tage/Woche, sechs Stunden/Tag) und somit kein unterschiedlicher physischer Stress gegeben war, ist es denkbar, dass ein durch den Geruch von Acetoin ausgelöster Fluchtreflex zu einer Stressreaktion geführt hat. Acetoin stellt einen Geruchsbestandteil von Katzenfäzes dar (Banik et al. 2021). Das Auftreten veränderter Blutparameter in der NTP-Studie (2023) nur bei den männlichen Tieren könnte darauf zurückzuführen sein, dass weibliche Ratten unempfindlicher gegen Gerüche oder immunologisch weniger sensibel reagieren. Die Abnahme an Lymphozyten in der Studie ist daher nicht direkt substanzbedingt. Die deutliche Zunahme des prozentualen Anteils an PCE bei der höchsten Konzentration weist auf eine gestörte Erythropoese hin.

**Fazit**: In der Gesamtbetrachtung passen die veränderten Blutwerte der männlichen Ratten der Studie des NTP (2023) zu dem von Everds et al. (2013) beschriebenen Stress-Leukogramm und werden daher nicht als advers angesehen. Die höchste Konzentration (800 ml/m^3^) wird als LOAEC bewertet, da hier erste Effekte in der Lunge, dem Larynx und den Nieren beobachtet wurden. Eine bronchioalveoläre Lavage zur Erkennung früher Entzündungsmarker im Atemtrakt wurde nicht durchgeführt. Obwohl bei den Ratten der 400-ml/m^3^-Konzentrationsgruppe keine histopathologische Untersuchung vorgenommen wurde, wird aufgrund der geringen Anzahl und minimalen Ausprägung der bei 800 ml/m^3^ aufgetretenen Effekte von einer NOAEC von 400 ml/m^3^ ausgegangen.

**Tab. 2:** Toxizität von Acetoin nach wiederholter inhalativer Exposition

**Spezies,** **Stamm, ** **Anzahl pro Gruppe**	**Exposition**	**Befunde^a)^**	**Literatur**
Ratte, Wistar, 5 ♀	14 d, 0; 6,25; 25; 100; 400; 800 ml/m^3^, 6 h/d, 5 d/Wo, Ganzkörper	**800 ml/m^3^**: **NOAEC**	NTP 2023
Ratte, Wistar, 10 ♂, 10 ♀, MN 5 ♂, 5 ♀	3 Mo, 0, 50, 100, 200, 400, 800 ml/m^3^, 6 h/d, 5 d/Wo, Ganzkörper, Blutuntersuchung am 4., 23., 93. Tag	**ab 50 ml/m^3^**: 23. Tag: ♂: Neutrophile ↓ (auf 55,7 % d. K.) nicht konzentrationsabhängig, bei Betrachtung der absoluten Zahlen: Neutrophile unauffällig (Tabelle 6), 93. Tag: alle Werte unauffällig; **ab 100 ml/m^3^**: 23. Tag: ♂: Hämatokritwert ↓ (auf 92,7 % d. K.), Hämoglobin ↓ (auf 92,3 % d. K.), beide Werte bei Betrachtung der absoluten Zahlen unauffällig, 93. Tag: alle Werte unauffällig; **200 ml/m^3^**: 93. Tag: ♂: Leukozyten ↓ (auf 81,6 % d. K.), Lymphozyten ↓ (auf 78,0 % d. K.), % PCE ↑ (um ca. 34 % d. K.); **400 ml/m^3^**: **NOAEC,** 93. Tag: ♂: Leukozyten ↓ (auf 82,4 % d. K.), Lymphozyten ↓ (auf 77,5 % d. K.), Monozyten ↓ (auf 71,6 % d. K.), % PCE ↑ (um ca. 35 % d. K.); **800 ml/m^3^**: 93. Tag: ♂: Leukozyten ↓ (auf 77,4 % d. K.), Lymphozyten ↓ (auf 70,9 % d. K.), Monozyten ↓ (auf 74,6 % d. K.), Eosinophile ↓ (auf 69 % d. K., n. s.), % PCE ↑ (um ca. 56 % d. K.), rel. Lebergewicht ↑ (um 7 %); nicht statistisch signifikante Effekte: Lunge: ♂: Histiozyten-Infiltration (2/10, minimal, Kontrolle 0/10), Metaplasie (1/10, minimal, Kontrolle 0/10), ♀: Entzündung (1/10, minimal, Kontrolle 0/10), Larynx: Entzündung (♂: 3/10 minimal–gering, Kontrolle: 1/10 minimal, ♀: 1/10 minimal, Kontrolle 0/10), Plattenepithelerosion (♂: 1/10 minimal, Kontrolle 0/10), Nieren: Nephropathie (♂: 3/10 minimal, Kontrolle: 2/10 minimal), Nierenbecken-Dilatation und chronische Entzündung und Übergangsepithel-Hyperplasie (♀: 1/10, Kontrolle 0/10)	NTP 2023
Maus, B6C3F1, 5 ♀	14 d, 0; 6,25; 25; 100; 400; 800 ml/m^3^, 6 h/d, 5 d/Wo, Ganzkörper	**800 ml/m^3^**: **NOAEC**	NTP 2023
Maus, B6C3F1, 10 ♂, 10 ♀	3 Mo, 0, 50, 100, 200, 400, 800 ml/m^3^, 6 h/d, 5 d/Wo, Ganzkörper	**800 ml/m^3^**: **NOAEC**	NTP 2023

a) wenn nicht anders angegeben, sind die aufgeführten Veränderungen statistisch signifikant

d: Tag; d. K.: des Kontrollwertes; h: Stunde; MN: Mikronukleustest; Mo: Monat; n. s.: nicht statistisch signifikant; PCE: polychromatische Erythrozyten; rel.: relativ; Wo: Woche

**Tab. 3:** Lymphozyten (× 10^3^/µl) nach Exposition gegen Acetoin, männliche Ratte, Mittelwerte ± SEM (NTP 2023)

**Expositionstag**	**0 ml/m^3^**	**50 ml/m^3^**	**100 ml/m^3^**	**200 ml/m^3^**	**400 ml/m^3^**	**800 ml/m^3^**
Tag 4	6,34 ± 0,35	7,02 ± 0,62	7,37 ± 0,89	6,03 ± 0,36	6,03 ± 0,71	8,23 ± 0,84
Tag 23	6,60 ± 0,35	7,00 ± 0,23	6,94 ± 0,29	6,96 ± 0,38	6,92 ± 0,49	7,57 ± 0,41
Tag 93	6,26 ± 0,35^##^	6,52 ± 0,40	5,61 ± 0,43	4,88 ± 0,24**	4,85 ± 0,35*	4,44 ± 0,24**

* p ≤ 0,05;

** p ≤ 0,01, paarweiser Vergleich

## p ≤ 0,01, Trendtest

SEM: Standardfehler des Mittelwertes

**Tab. 4:** Leukozyten (× 10^3^/µl) nach Exposition gegen Acetoin, männliche Ratte, Mittelwerte ± SEM (NTP 2023)

**Expositionstag**	**0 ml/m^3^**	**50 ml/m^3^**	**100 ml/m^3^**	**200 ml/m^3^**	**400 ml/m^3^**	**800 ml/m^3^**
Tag 4	7,18 ± 0,36	8,13 ± 0,66	8,36 ± 0,99	7,07 ± 0,39	7,02 ± 0,78	9,45 ± 0,93
Tag 23	8,33 ± 0,35	8,10 ± 0,27	7,92 ± 0,35	8,03 ± 0,38	8,13 ± 0,55	8,54 ± 0,44
Tag 93	7,61 ± 0,41^##^	8,06 ± 0,37	7,00 ± 0,47	6,21 ± 0,30*	6,27 ± 0,48*	5,89 ± 0,24**

* p ≤ 0,05;

** p ≤ 0,01, paarweiser Vergleich

# p ≤ 0,01, Trendtest

SEM: Standardfehler des Mittelwertes

**Tab. 5:** Neutrophilenzahlen (× 10^3^/µl) bei männlichen Wistar-Kontroll-Ratten (n = 10) in NTP-Studien, Mittelwerte ± SEM

**Expositionstag**	**Acetoin (NTP 2023)**	**2,3-Pentandion (NTP 2023)**	**Diacetyl (NTP 2018)**
Tag 4	0,56 ± 0,03	1,11 ± 0,12	1,03 ± 0,11
Tag 23	1,49 ± 0,17	1,31 ± 0,17	1,22 ± 0,09
Tag 93	1,10 ± 0,11	1,25 ± 0,07	1,47 ± 0,21

SEM: Standardfehler des Mittelwertes

**Tab. 6:** Neutrophilenzahlen (× 10^3^/µl) nach Exposition gegen Acetoin, männliche Ratte, Mittelwerte ± SEM (NTP 2023)

**Expositionstag**	**0 ml/m^3^**	**50 ml/m^3^**	**100 ml/m^3^**	**200 ml/m^3^**	**400 ml/m^3^**	**800 ml/m^3^**
Tag 4	0,56 ± 0,03	0,75 ± 0,06	0,70 ± 0,09	0,73 ± 0,08	0,71 ± 0,08	0,88 ± 0,09
Tag 23	1,49 ± 0,17^#^	0,83 ± 0,06*	0,76 ± 0,12**	0,83 ± 0,06*	0,92 ± 0,08	0,75 ± 0,05**
Tag 93	1,10 ± 0,11	1,27 ± 0,11	1,18 ± 0,07	1,14 ± 0,11	1,23 ± 0,09	1,27 ± 0,05

* p ≤ 0,05;

** p ≤ 0,01, paarweiser Vergleich. Die statistische Signifikanz ist vermutlich auf die sehr hohen Neutrophilenzahlen der Kontrolltiere zurückzuführen.

# p ≤ 0,05, Trendtest. Die statistische Signifikanz ist vermutlich auf die sehr hohen Neutrophilenzahlen der Kontrolltiere zurückzuführen.

SEM: Standardfehler des Mittelwertes

#### 5.2.2 Orale Aufnahme

Je 15 männliche und weibliche CFE-Ratten erhielten 13 Wochen lang Acetoin im Trinkwasser in Konzentrationen von 0, 750, 3000 oder 12 000 mg/l (entspricht bei den männlichen Tieren 0, 80, 318 oder 1286 mg/kg KG und Tag und bei den weiblichen Tieren 0, 91, 348 oder 1404 mg/kg KG und Tag). Nur in der höchsten Dosisgruppe traten statistisch signifikante Veränderungen auf: Das Körpergewicht der männlichen Tiere nahm ab der 5. Woche ab und das relative Lebergewicht war bei den männlichen Tieren ab der zweiten Woche sowie bei weiblichen Ratten nach 13 Wochen erhöht (k. w. A.). Die hämatologische Untersuchung nach 13 Wochen zeigte eine geringe (4–8 %), aber statistisch signifikante (p < 0,05) Abnahme der Hämoglobinkonzentration und der Erythrozytenzahl bei den Tieren beider Geschlechter, wobei diese Veränderungen nicht mit einer Abnahme des Hämatokritwertes einhergingen. Nach sechs Wochen, allerdings nicht am Ende der Studie, waren auch die Leukozyten erniedrigt, jedoch nur bei den weiblichen Tieren statistisch signifikant. Die Untersuchungen klinisch-chemischer Parameter und der Histologie ergaben keine statistisch signifikanten Unterschiede zwischen den behandelten Tieren und den Kontrollgruppen. Trotz der hohen Dosis verendete kein Tier. Die erhöhten relativen Lebergewichte bei beiden Geschlechtern könnten eine Reaktion der Leber auf eine erhöhte Stoffwechselbelastung durch die hohe Acetoinaufnahme gewesen sein. Der NOAEL betrug 318 bzw. 348 mg/kg KG und Tag (Gaunt et al. 1972).

#### 5.2.3 Dermale Aufnahme

Hierzu liegen keine Daten vor.

### 5.3 Wirkung auf Haut und Schleimhäute

#### 5.3.1 Haut

Unter okklusiven Bedingungen war die unverdünnte Substanz nach 24-stündiger Applikation auf intakter und skarifizierter Kaninchenhaut mäßig reizend (k. w. A.; Opdyke 1979).

Nach 24-stündiger Inkubation von EpiDerm^TM^-Gewebe mit 1 mg Acetoin/ml und 24-stündiger Nachbeobachtungszeit fand sich noch eine Viabilität des Gewebes von 94,3 % und damit ist im RhE-Test-System keine Reizwirkung beobachtet worden (ECHA 2019).

Acetoin ist nach dem global harmonisierten System von den REACH-Registranten nicht in eine Kategorie für hautreizende Stoffe eingestuft (ECHA 2019).

#### 5.3.2 Auge

Im nach OECD-Prüfrichtlinie 437 durchgeführten In-vitro-Test verursachte eine 10-minütige Exposition von 750 µl unverdünntem Acetoin schwere Schäden an der Hornhaut des Rinderauges. Der In-vitro-Reizindex (IVIS) betrug ca. 121,82. Die Tiere waren bei Entnahme der Augen zwischen 12 und 60 Monate alt (ECHA 2019). Ab einem IVIS > 55 wird eine Einstufung in Kategorie 1 für augenreizende Stoffe vorgenommen.

In den Inhalationsstudien wurden keine Augenschäden bei Ratten und Mäusen festgestellt (NTP 2023).

Acetoin ist nach dem global harmonisierten System von den REACH-Registranten in die Kategorie 1 für augenreizende Stoffe eingestuft (ECHA 2019).

#### 5.3.3 Fazit

Acetoin wirkt an der Kaninchenhaut nach 24-stündiger okklusiver Applikation mäßig reizend, im RhE-Test in vitro nicht reizend. Der Stoff verursacht in vitro schwere Augenschäden.

### 5.4 Allergene Wirkung

#### 5.4.1 Hautsensibilisierende Wirkung

##### 5.4.1.1 Tierexperimentelle Untersuchungen

Es liegen keine tierexperimentellen Untersuchungen vor.

##### 5.4.1.2 Untersuchungen mit tierversuchsfreien Alternativmethoden (NAMs)

Zur Ableitung hautsensibilisierender Eigenschaften einer Chemikalie werden gemäß der OECD-Richtlinie 497 die Ergebnisse aus Testverfahren, die Schlüsselereignisse des Adverse Outcome Pathway (AOP) prüfen, miteinander verknüpft. Die Berücksichtigung mehrerer Testverfahren und deren Verknüpfung bei der Ableitung des sensibilisierenden Potentials einer Chemikalie beruht auf der Annahme, dass ein Einzeltestverfahren die komplexe Abfolge bei der Entwicklung einer Sensibilisierung nicht abbilden kann.

Die experimentellen OECD-validierten Testverfahren basieren auf dem AOP für Sensibilisierung (OECD 2012). Methoden zur Prüfung des ersten Schlüsselereignisses (key event 1, KE1; und molecular initiating event, MIE) testen die Bindungsfähigkeit an Hautproteine (elektrophile-nukleophile Interaktion). Das zweite Schlüsselereignis (KE2) bzw. entsprechende Prüfmethoden analysieren eine substanzinduzierte Aktivierung von Keratinozyten. Als drittes Schlüsselereignis (KE3) wird die substanzinduzierte Reifung von dendritischen Zellen getestet. Zur Prüfung des vierten Schlüsselereignisses (KE4), die T-Zell-Proliferation, gibt es derzeit noch kein validiertes Testverfahren. Neben der experimentellen Testung der verschiedenen KEs, die auf In-chemico-Ansätzen (KE1) und In-vitro-Verfahren (KE2–KE3) basieren, können auch zusätzlich Daten aus Modellen (in silico) zur Bewertung herangezogen werden, sowie der Wirkungsmechanismus betrachtet werden.

Für Acetoin liegen Ergebnisse für das erste und zweite Schlüsselereignis vor.

###### 5.4.1.2.1 Schlüsselereignis 1 des AOP für Sensibilisierung: Prüfung der Substanz auf Peptidreaktivität 

Zur Prüfung der Substanz auf Peptidreaktivität stehen derzeit verschiedene OECD-validierte Methoden (Prüfrichtlinie 442C) zur Verfügung. Acetoin wurde im Direkten Peptidreaktivitätstest (DPRA) negativ getestet (ECHA 2019).

Das Ergebnis steht im Einklang mit Erwartungen aufgrund der chemischen Struktur von Acetoin (Roberts et al. 2007).

###### 5.4.1.2.2 Schlüsselereignis 2 des AOP für Sensibilisierung: Prüfung der Substanz auf substanzinduzierte Aktivierung von Keratinozyten 

Zur Testung des zweiten Schlüsselereignisses wird die substanzinduzierte Aktivierung von Keratinozyten bestimmt. Hierzu stehen momentan verschiedene OECD-validierte (Prüfrichtlinie 442D) sowie wissenschaftlich anerkannte Methoden zur Verfügung. Acetoin wurde im LuSens (ARE-Nrf2 Luciferase LuSens Test) negativ getestet (ECHA 2019).

Untersuchungen mit computergestützten Methoden liefern ebenfalls negative Ergebnisse (OECD Toolbox v4.2; Toxtree v3.1.0) (Api et al. 2022).

Zur Verknüpfung der vorliegenden Einzelergebnisse zu einem Gesamtergebnis wurden beispielhaft zwei Integrationsansätze in Anlehnung an OECD-Richtlinie 497 angewandt. Im ersten Ansatz, „2 of 3“-Ansatz, werden Daten aus Standard-DPRA, KeratinoSens und h-CLAT (Human Cell Line Activation Test) genutzt. Bei Erweiterung des Prinzips (anstelle des KeratinoSens wird der LuSens eingesetzt) und Berücksichtigung des negativen Ergebnisses im LuSens ist das Gesamtergebnis negativ.

Zur Ableitung eines Gesamtergebnisses unter Anwendung eines zweiten Ansatzes, der Integrated Testing Strategy Version 2 (ITSv2), gemäß der OECD-Richtlinie 497 können Ergebnisse aus dem DPRA (KE1), dem h-CLAT (KE3) sowie Ergebnisse aus Untersuchungen aus computergestützten Methoden (OECD QSAR Toolbox v 4.7) berücksichtigt werden. Für Acetoin liegt kein Ergebnis im h-CLAT vor. Daher basiert die Integration auf den Daten des DPRA (bewertet mit 0/3 Punkten) und der OECD QSAR Toolbox (0/1 Punkten). Insgesamt ergibt sich daraus eine Gesamtpunktzahl von 0/4 Punkten, was gemäß OECD-Richtlinie 497 zu einem inkonklusiven Gesamtergebnis bezüglich der hautsensibilisierenden Wirkung führt.

#### 5.4.2 Atemwegssensibilisierende Wirkung

Hierzu liegen keine Daten vor.

### 5.5 Reproduktionstoxizität

Hierzu liegen keine Daten vor.

### 5.6 Genotoxizität

#### 5.6.1 In vitro

Es liegen nur bakterielle Mutagenitätstests mit den Salmonella Stämmen TA97, TA98, TA100, TA1535, TA1537, TA1538 und E. coli WP2 uvrA mit und ohne Zusatz eines metabolischen Aktivierungssystems (S9-Mix) vor. Acetoin zeigte keine mutagene Wirkung. Die genauen Daten sind in Tabelle 7 angegeben (Aeschbacher et al. 1989; Kim et al. 1987; NTP 2023).

**Tab. 7:** Genotoxizität von Acetoin in vitro

**Endpunkt (Testmethode)**	**Testsystem**	**Konzentration**	**wirksame Konzentration**	**Zytotoxizität^a)^**	**Ergebnis^a)^**		**Literatur**
					–m. A.	+m. A.	
Genmutation	S. typhimurium TA97, TA98, TA100, TA1535	0, 100–10 000 µg/Platte	–	–	–	–	NTP 2023
	S. typhimurium TA100	k. A.	–	k. A.	–	–	NTP 2023
Genmutation (Präinkubation)	S. typhimurium TA98, TA100, TA102	0, 5–500 nmol/Platte	–	k. A.	–	–	Aeschbacher et al. 1989
Genmutation (Präinkubation)	S. typhimurium TA98, TA100, TA1535, TA1537, TA1538, E. coli WP2 uvrA	0, 1–5000 µg/Platte	–	k. A.	–	n. u.	EFSA CEF Panel 2014
	S. typhimurium TA100	4,44 µmol/Platte	–	k. A.	–	n. u.	Kim et al. 1987

a) Ergebnis statistisch signifikant, wenn nicht anders angegeben

–: negatives Ergebnis; k. A.: keine Angaben; m. A.: metabolische Aktivierung; n. u.: nicht untersucht

#### 5.6.2 In vivo

Nach dreimonatiger inhalativer Ganzkörperexposition von Ratten und Mäusen gegen 0, 50, 100, 200, 400 oder 800 ml Acetoin/m^3^ wurde ein Mikronukleustest mit den Zellen des peripheren Blutes durchgeführt. Ein erhöhtes Auftreten an Mikronuklei wurde nicht beobachtet. Die genauen Daten sind in Tabelle 8 angegeben. Ein konzentrationsabhängiger Anstieg ab 200 ml/m^3^ des Prozentsatzes unreifer polychromatischer Erythrozyten (% PCE) wurde bei männlichen, aber nicht bei weiblichen Ratten beobachtet (siehe Abschnitt 5.2.1; NTP 2023). Die Zunahme der PCE kann auf Stress, aber aufgrund des deutlichen Anstiegs bei der höchsten Konzentration auch auf eine leichte Störung der Erythropoese bei männlichen Ratten zurückgeführt werden. Die veränderte Erythropoese gibt einen Hinweis darauf, dass das Knochenmark erreicht wurde.

**Tab. 8:** In-vivo-Studien zur Genotoxizität von Acetoin

**Endpunkt **	**Testsystem**	**Exposition**	**Ergebnis^a)^**	**Zytotoxizität/Anmerkungen**	**Literatur**
MN, peripheres Blut	Maus, B6C3F1, je 5 ♂, ♀	90 d, 0, 50, 100, 200, 400, 800 ml/m^3^, Untersuchung nach 24 h	–	Reinheit: 99,4 %	NTP 2023
MN, peripheres Blut	Ratte, Wistar, je 5 ♂, ♀	90 d, 0, 50, 100, 200, 400, 800 ml/m^3^, Untersuchung nach 24 h	–	Reinheit: 99,4 %, ♂: ab 200 ml/m^3^ PCE/NCE ↑ (34 % mehr PCE im Blut), bei 800 ml/m^3^ 56 % mehr PCE im Blut	NTP 2023

a) Ergebnis statistisch signifikant, wenn nicht anders angegeben

–: negatives Ergebnis; d: Tag; h: Stunde; MN: Mikronukleustest; NCE: normochromatische Erythrozyten; PCE: polychromatische Erythrozyten

### 5.7 Kanzerogenität

#### 5.7.1 Kurzzeitstudien

Je 20–30 weiblichen A/He-Mäusen wurden sieben Wochen lang dreimal pro Woche 0; 0,6 oder 3 g Acetoin/kg KG, in Wasser gelöst, intraperitoneal injiziert. Die Dosen entsprachen einem Fünftel der maximal tolerablen Dosis (MTD) bzw. der MTD. Die MTD wurde in einem Vorversuch mit fünf Mäusen nach sechs Injektionen innerhalb von zwei Wochen und einer ein- bis zweimonatigen Nachbeobachtungszeit ermittelt. Nach 22 Wochen wurden keine erhöhten Inzidenzen für Lungentumore beobachtet (Stoner et al. 1973). Dieser Versuchsansatz galt als Screening-Test auf Kanzerogenität und wurde mit besonders lungentumorempfindlichen Mäusen durchgeführt.

#### 5.7.2 Langzeitstudien

Hierzu liegen keine Daten vor.

### 5.8 Sonstige Wirkungen

Die Behandlung mit 100 µM Acetoin induzierte die Freisetzung von IL-8 in einer humanen bronchialen Epithelzelllinie (Beas2B-Zellen). Mit 1 mM Acetoin behandelte humane primäre Lungenfibroblasten (HFL-1-Zellen) reagierten mit einer leicht erhöhten Freisetzung von IL-8 im Vergleich zur Kontrolle. Es wurde jedoch nicht der Wert wie mit TNF-α, das als Positivkontrolle eingesetzt wurde, erreicht. Bei humanen transformierten Lungenepithelzellen (H292) konnte keine IL-8 Freisetzung durch Acetoin hervorgerufen werden. Die Autoren verweisen auf das proinflammatorische Potential von Acetoin und weiteren Inhaltsstoffen von E-Liquids (Gerloff et al. 2017).

## 6 Bewertung

Das grundlegende Vorgehen zur Bewertung eines Arbeitsstoffes ist der MAK- und BAT-Werte-Liste zu entnehmen (DFG 2025).

Kritische Effekte sind Entzündungen im Respirationstrakt und eine augenreizende Wirkung.

**MAK-Wert. **Nach dreimonatiger inhalativer Exposition treten bei der höchsten Konzentration von 800 ml Acetoin/m^3^ bei männlichen und in geringerem Umfang bei den weiblichen Ratten Entzündungen mit minimalen bis geringen Ausprägungen in Larynx, Lunge und Nieren auf. Eine unmittelbare Reizwirkung in den Atemwegen stand hier jedoch nicht im Vordergrund und auch Augenreizungen wurden nicht festgestellt (NTP 2023). Die NOAEC für entzündliche Prozesse im Atemtrakt beträgt in dieser Inhalationsstudie 400 ml/m^3^ (Abschnitt 5.2.1). Die aus dem Ergebnis des In-vitro-Tests am Rinderauge gefolgerte starke lokale Reizwirkung hat sich damit nach dreimonatiger Ganzkörperexposition bei Ratten und Mäusen nicht bestätigt.

Damit errechnet sich aus der NOAEC von 400 ml/m^3^ mit der Annahme einer Verstärkung der Effekte bei chronischer Exposition (1:2), mit Übertragung der Daten aus dem Tierversuch auf den Menschen (1:2) und der Berücksichtigung des erhöhten Atemvolumens (1:2) ein MAK-Wert von 50 ml/m^3^.

**Spitzenbegrenzung. **In der Inhalationsstudie trat keine unmittelbare Reizwirkung im respiratorischen System auf, daher wird Acetoin in die Spitzenbegrenzungs-Kategorie II eingestuft. Genaue Daten zur Halbwertszeit von Acetoin fehlen. Aus diesem Grund erfolgt die Spitzenbegrenzung mit dem Standard-Überschreitungsfaktor von 2.

**Fruchtschädigende Wirkung. **Es liegen keine Daten zur Entwicklungstoxizität und auch kein Verdacht auf eine entwicklungstoxische Wirkung (Gruppe B (Verdacht)) vor.

Acetoin wird der Schwangerschaftsgruppe D zugeordnet.

**Krebserzeugende Wirkung. **Acetoin ist nicht mutagen in bakteriellen Mutagenitätstests und zeigt keine genotoxischen Effekte in vivo im Mikronukleustest bei Ratte und Maus. In einer Kurzzeitstudie mit inhalativer Exposition sind nach intraperitonealer Verabreichung bei Mäusen keine erhöhten Lungentumorinzidenzen aufgetreten. Da Acetoin kein Diketon ist, wie die weiteren Butteraromastoffe Diacetyl und 2,3-Pentandion, liegt hier auch kein Strukturverdacht vor.

Damit erfolgt keine Einstufung in eine Kanzerogenitäts-Kategorie.

**Keimzellmutagene Wirkung. **Acetoin ist nicht mutagen in bakteriellen Mutagenitätstests und zeigt keine genotoxischen Effekte in vivo im Mikronukleustest bei Ratte und Maus nach 90-tägiger inhalativer Exposition. Studien an Keimzellen liegen nicht vor.

Damit erfolgt keine Einstufung in eine Kategorie für Keimzellmutagene.

**Hautresorption. **Zur perkutanen Resorption von Acetoin liegen keine experimentellen Daten vor. Für den Menschen lassen sich über Modellrechnungen (Abschnitt 3.1) mit den zwei angegebenen log K_OW_ dermale Aufnahmemengen von 895–7509 mg bei Exposition gegen eine gesättigte wässrige Lösung unter Standardbedingungen (2000 cm^2^ Hautoberfläche, eine Stunde Exposition) abschätzen.

Die systemische subchronische NOAEC nach Inhalation bei Ratten beträgt 400 ml/m^3^ (1480 mg/m^3^). Zur toxikokinetischen Übertragung dieser Konzentration als systemischen NOAEL auf den Menschen werden berücksichtigt: das Atemvolumen in acht Stunden (10 m^3^), die angenommene 100%ige inhalative Resorption, eine mögliche Wirkungsverstärkung mit der Zeit (1:2), die Übertragung der Daten des Tierversuchs auf den Menschen (1:2) und das erhöhte Atemvolumen am Arbeitsplatz (1:2). Damit errechnet sich eine systemisch tolerable Menge von 1850 mg.

Der orale systemische subchronische NOAEL bei männlichen Ratten beträgt 318 mg/kg KG. Zur toxikokinetischen Übertragung dieser Dosis als systemischen NOAEL auf den Menschen werden berücksichtigt: der dem toxikokinetischen Unterschied zwischen Ratte und dem Menschen entsprechende speziesspezifische Korrekturwert (1:4), die angenommene orale Resorption von 100 %, die tägliche Exposition der Tiere im Vergleich zur fünftägigen Exposition pro Woche am Arbeitsplatz (7:5), das Körpergewicht (70 kg) des Menschen, eine mögliche Wirkungsverstärkung mit der Zeit (1:2) und die Übertragung der Daten des Tierversuchs auf den Menschen (1:2). Damit errechnet sich eine systemisch tolerable Menge von 1948 mg.

Die systemisch tolerable Menge beträgt daher ca. 1900 mg.

Damit liegt die Aufnahme über die Haut bei allen Berechnungsansätzen bei deutlich mehr als 25 % der systemisch tolerablen Menge, und der Stoff wird mit „H“ markiert.

**Sensibilisierende Wirkung. **Es liegen keine Hinweise auf eine hautsensibilisierende Wirkung von Acetoin beim Menschen vor. Tierexperimentelle Untersuchungen liegen nicht vor. Daten aus tierversuchsfreien Alternativverfahren liefern in Anlehnung an OECD-Richtlinie 497 ebenfalls keine Hinweise auf eine hautsensibilisierende Wirkung. Aufgrund der chemischen Struktur besteht kein Verdacht auf eine sensibilisierende Wirkung. Zur atemwegssensibilisierenden Wirkung von Acetoin liegen keine Daten vor. Acetoin wird daher weder mit „Sh“, noch mit „Sa“ markiert.
